# Nuclear translocation of the cytoplasmic domain of HB-EGF induces gastric cancer invasion

**DOI:** 10.1186/1471-2407-12-205

**Published:** 2012-05-30

**Authors:** Takaya Shimura, Michihiro Yoshida, Shinji Fukuda, Masahide Ebi, Yoshikazu Hirata, Tsutomu Mizoshita, Satoshi Tanida, Hiromi Kataoka, Takeshi Kamiya, Shigeki Higashiyama, Takashi Joh

**Affiliations:** 1 Department of Gastroenterology and Metabolism, Nagoya City University Graduate School of Medical Sciences, 1 Kawasumi, Mizuho-cho, Mizuho-ku, Nagoya, 467-8601, Japan; 2 Department of Biochemistry and Molecular Genetics, Ehime University Graduate School of Medicine, Shitsukawa, Toon, Ehime, 791-0295, Japan; 3 Department of Cell Growth and Tumor Regulation, Proteo-Medicine Research Center, Ehime University, Shitsukawa, Toon, Ehime, 791-0295, Japan

**Keywords:** EGFR, Gastric cancer, HB-EGF, Cancer invasion

## Abstract

**Background:**

Membrane-anchored heparin-binding epidermal growth factor-like growth factor (proHB-EGF) yields soluble HB-EGF, which is an epidermal growth factor receptor (EGFR) ligand, and a carboxy-terminal fragment of HB-EGF (HB-EGF-CTF) after ectodomain shedding. We previously reported that HB-EGF-CTF and unshed proHB-EGF which has the cytoplasmic domain of proHB-EGF (HB-EGF-C), translocate from the plasma membrane to the nucleus and regulate cell cycle after shedding stimuli. However, the significance of nuclear exported HB-EGF-C in human gastric cancer is unclear.

**Methods:**

We investigated the relationship between intracellular localization of HB-EGF-C and clinical outcome in 96 gastric cancer patients treated with gastrectomy. Moreover, we established stable gastric cancer cell lines overexpressing wild-type HB-EGF (wt-HB-EGF) and mutated HB-EGF (HB-EGF-mC), which prevented HB-EGF-C nuclear translocation after shedding. Cell motility between these 2 gastric cancer cell lines was investigated using a transwell invasion assay and a wound healing assay.

**Results:**

Of the 96 gastric cancer cases, HB-EGF-C immunoreactivity was detected in both the nucleus and cytoplasm in 19 cases (19.8 %) and in the cytoplasm only in 25 cases (26.0 %). The nuclear immunoreactivity of HB-EGF-C was significantly increased in stage pT3/4 tumors compared with pT1/2 tumors (T1/2 vs. T3/4: 11.1 % vs. 36.4 %, *P* < 0.01). The growth of wt-HB-EGF- and HB-EGF-mC-expressing cells significantly increased compared with control cells, but the growth of HB-EGF-mC-expressing cells was significantly decreased compared with wt-HB-EGF-expressing cells. Gastric cancer cell invasion obviously increased in wt-HB-EGF-expressing cells, but invasion in HB-EGF-mC-expressing cells showed a slight increase compared with control cells. Moreover, wt-HB-EGF overexpression increased the effectiveness of wound healing, but had no significant effect in HB-EGF-mC-expressing cells.

**Conclusions:**

Both the function of HB-EGF as an EGFR ligand and a novel signal for HB-EGF-C nuclear translocation induce gastric cancer growth, whereas HB-EGF-C nuclear translocation independently plays a critical role in gastric cancer invasion. The present study demonstrated that HB-EGF-C nuclear translocation might be crucial in gastric cancer invasion. HB-EGF-C nuclear translocation may offer a prognostic marker and a new molecular target for gastric cancer therapy.

## Background

Gastric cancer is the fourth most common malignancy and the second leading cause of cancer death in the world [1]. Despite the recent development of several novel cytotoxic agents, the prognosis of advanced gastric cancer remains poor, and new treatments that show acceptable toxicity profiles are urgently needed. No molecular target agent has shown sufficient clinical effects in gastric cancer, but trastuzumab, a monoclonal antibody that targets human epidermal growth factor receptor 2 (HER2; also known as ERBB2), was recently shown in a global phase III clinical trial to confer a survival benefit for HER2-positive advanced gastric cancer [2]. Although this molecular target agent was clinically approved for use in gastric cancer therapy, further development of biomarkers should be pursued because only approximately 20 % of all gastric cancers expressing HER-2. The epidermal growth factor receptor (EGFR) belongs to the ErbB receptor tyrosine kinase family, which includes erbB1 (EGFR), erbB2 (HER2), erbB3 (HER3), and erbB4 (HER4). EGFR plays a key role in cancer regulation. EGFR has 7 known ligands, all of which are synthesized as type I transmembrane protein precursors and are subsequently expressed on the plasma membrane [3]. The increased expression of EGFR ligands, including transforming growth factor TGF-α, heparin-binding EGF-like growth factor (HB-EGF), and amphiregulin, is associated with clinical prognosis in many cancers such as gastric cancer [4,5].

**Figure 1:**
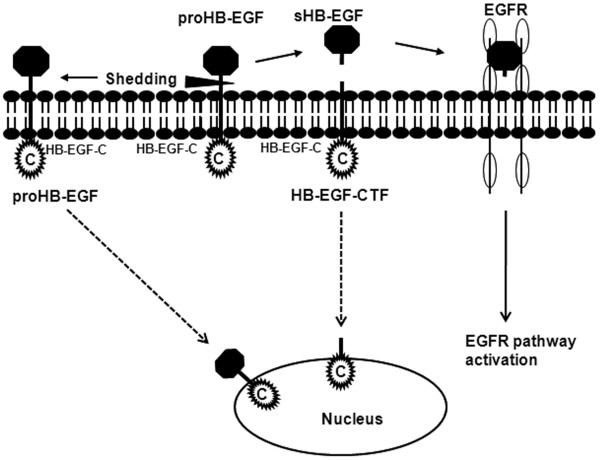
**Nuclear translocation of HB-EGF-C.** Membrane-anchored heparin-binding epidermal growth factor-like growth factor (proHB-EGF) yields soluble HB-EGF (sHB-EGF) and a carboxy-terminal fragment of HB-EGF (HB-EGF-CTF) after ectodomain shedding. sHB-EGF binds to EGFR as a ligand and activates the downstream signal pathways. HB-EGF-CTF translocates from plasma membrane to the nucleus after shedding and the cytoplasmic domain of HB-EGF (HB-EGF-C) binds to some transcriptional repressors in the nucleus. Moreover, unshed proHB-EGF translocates to the nucleus after shedding stimuli. HB-EGF-C is included in both HB-EGF-CTF and proHB-EGF.

Membrane-anchored HB-EGF (proHB-EGF), an EGFR ligand, is cleaved from the plasma membrane in a process termed ectodomain shedding, which yields soluble HB-EGF (s-HB-EGF) and a carboxy-terminal fragment of HB-EGF (HB-EGF-CTF) (Figure 1). s-HB-EGF binds to EGFR and induces activation of intracellular signaling cascades that are implicated in the regulation of a wide variety of cellular processes, including growth, differentiation, apoptosis, adhesion, and migration. We previously showed HB-EGF-CTF translocates from plasma membrane to the nucleus after ectodomain shedding of proHB-EGF and regulates cyclin A and cyclin D2 by the cytoplasmic domain of proHB-EGF (HB-EGF-C) binding to transcriptional repressors, such as promyelocytic leukemia zinc finger (PLZF) and B-cell lymphoma 6 (Bcl6) [6,7]. We previously showed that BCL6 expression is linked to the downregulation of cyclin D2 in HB-EGF-positive human gastric cancer cells [8]. Moreover, we have shown *in vitro* that suppression of HB-EGF-CTF nuclear translocation may be a new molecular target for gastric cancer therapy [9]. Subsequently, we showed that not only HB-EGF-CTF but also unshed proHB-EGF translocate to the nucleus after exposure to shedding stimuli and HB-EGF-C is responsible for various functions [10]. However, the details of how HB-EGF-C actually works in human gastric cancer remain unclear. Thus, we analyzed the relationship between expression and localization of HB-EGF and clinical behavior by using human gastric cancer specimens. Furthermore, we constructed mutated HB-EGF at the C-terminus (HB-EGF-mC), which did not translocate to the nucleus after shedding [10], and 2 gastric cancer cell lines that stably overexpressed full-length HB-EGF and HB-EGF-mC. We verified the significance of HB-EGF-C in gastric cancer by using these gastric cancer cell lines.

We herein demonstrate that the nuclear translocation of HB-EGF-C is critical for the invasion and progression of human gastric cancer.

## Results

### Patient Characteristics

Characteristics of patients in this study are shown in Table 1. Subjects included 96 patients, with 59 men and 37 women. The median age was 69 years (range, 37–91 years), and histological types included intestinal type in 59 patients and diffuse type in 37 patients. According to the Union for International Cancer Control (UICC) tumor-node-metastasis (TNM) classification, the stage of primary tumor was pT1 in 19 cases, pT2 in 44 cases, pT3 in 22 cases, and pT4 in 11 cases.

**Table 1:** Characteristics of gastric cancer patients who underwent gastrectomy

**Total no. of patients**		**96**
**Gender**	**Male**	**59**
	**Female**	**37**
**Age (years)**	**Median**	**69**
	**(range)**	**(37–91)**
**Histological type**	**Intestinal type**	**59**
	**Diffuse type**	**37**
**Stage of primary tumor**	**(pT1/pT2/pT3/pT4)**	**(19/44/22/11)**
**(UICC-TNM)**		

UICC-TNM, Seventh edition of the Union for International Cancer Control tumor-node-metastasis classification.

### HB-EGF-C expression in human gastric cancer

**Figure 2:**
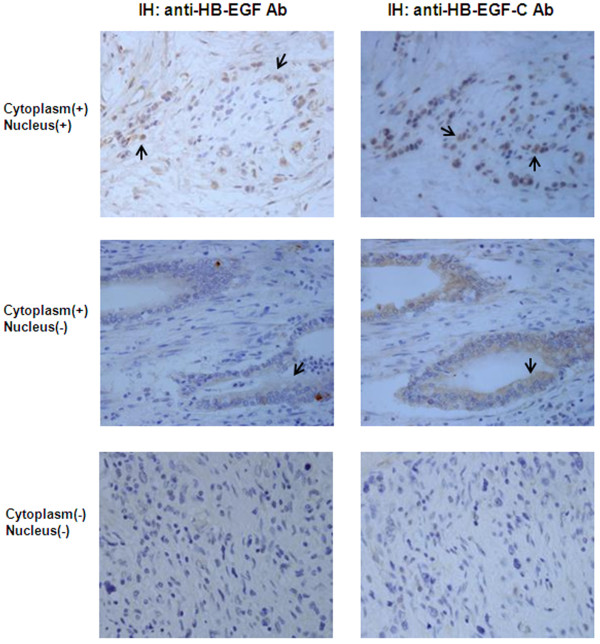
**Immunohistochemistry in human gastric cancer.** Heparin-binding epidermal growth factor-like growth factor (HB-EGF) expression was immunohistochemically investigated using samples of surgically resected gastric cancer cells. Cells were immunostained using anti-HB-EGF antibody and anti-HB-EGF-C antibody. Representative positive staining cells were shown by black arrows. (Upper pictures) HB-EGF and HB-EGF-C were detected in the cytoplasm and nucleus of gastric cancer cells. (Middle pictures) HB-EGF and HB-EGF-C were detected in only the cytoplasm of gastric cancer cells. (Lower pictures) HB-EGF and HB-EGF-C were not detected in gastric cancer cells. (Original magnification: ×400).

Immunoreactivity for HB-EGF-C was categorized into 3 patterns: staining of nucleus and cytoplasm for HB-EGF-C, staining of only the cytoplasm for HB-EGF, or no staining (Figure 2). Intracellular localization for HB-EGF was consistent with that of HB-EGF-C in all cases, but the nuclear immunoreactive rate for HB-EGF was clearly low compared with HB-EGF-C. Hence, HB-EGF-C immunoreactivity in the cytoplasm reflected proHB-EGF expression and HB-EGF-C immunoreactivity in the nucleus reflected nuclear localization of HB-EGF-CTF and proHB-EGF.

The immunohistochemical analysis of HB-EGF-C in human gastric cancer according to clinical stage is shown in Table 2. Of the 96 gastric cancer cases examined, HB-EGF-C staining was detected in 44 cases (45.8 %). Among the 44 cases with HB-EGF-C-positive staining, 25 cases (26.0 %) exhibited staining in both the nucleus and in the cytoplasm; however, 19 cases (19.8 %) exhibited staining only in the cytoplasm.

**Table 2:** HB-EGF-C expression and localization according to clinical stage

**Expression**	(+)*		**(−)**	
**Localization**	**C (+) N (+)**	**C (+) N (−)**	**C (−) N (−)**	**Total (n)**
**pT1, 2**	7 (11.1)**	**15 (23.8)**	**41 (65.1)**	**63**
**n, (%)**				
**pT3, 4**	12 (36.4)**	**10 (30.3)**	**11 (33.3)**	**33**
**n, (%)**				
**Total**	**19 (19.8)**	**25 (26.0)**	**52 (54.2)**	**96**
**n, (%)**				

C, cytoplasm; N, nucleus; (+), positive staining; (−), negative staining.

* *P* < 0.01, pT3, 4 vs. pT1, 2 for the rate of C (+) N (+) and C (+) N (−).

** *P* < 0.01, pT3, 4 vs. pT1, 2 for the rate of C (+) N (+).

Of 63 cases with pT1 and pT2, HB-EGF-C immunoreactivity was detected in 7 cases (11.1 %) in the nucleus and the cytoplasm and in 15 (23.8 %) in the cytoplasm only, but it was not observed in 41 cases (65.1 %). However, among 33 cases with pT3 and pT4, 12 (36.4 %) showed HB-EGF-C staining in the nucleus and in the cytoplasm, 10 (30.3 %) showed staining in the cytoplasm only, and 11 (33.3 %) did not exhibit immunoreactivity for HB-EGF-C. HB-EGF-C expression was significantly increased in cases with stage pT3 and pT4 compared to those with pT1 and pT2 (*P* < 0.01), and nuclear expression for HB-EGF-C was also significantly increased in cases with stage pT3 and pT4 (*P* < 0.01). These results suggest that not only proHB-EGF expression, but also nuclear translocation of proHB-EGF and HB-EGF-CTF may play crucial roles in gastric cancer invasion.

### Expression and intracellular localization of HB-EGF-C in each gastric cancer cell line

**Figure 3:**
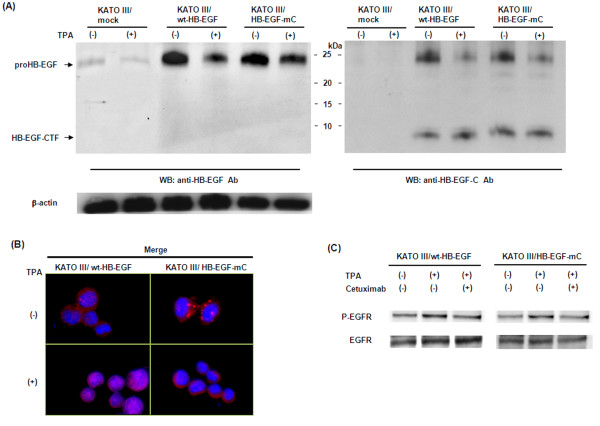
**Characteristics of KATO III/wt-HB-EGF and KATO III/HB-EGF-mC cells. A**) Western blot analysis of proHB-EGF and HB-EGF-CTF expression in 3 gastric cancer cell lines. Anti-HB-EGF antibody was used to recognize the proHB-EGF ectodomain and anti-HB-EGF-C antibody was used to recognize the cytoplasmic region of proHB-EGF. Each lane contains 100 μg of protein. Cleavage of proHB-EGF was stimulated using 200 nM TPA. **B**) HB-EGF-C localization after TPA-inducible processing of proHB-EGF in KATO III/wt-HB-EGF and KATO III/HB-EGF-mC cells by immunofluorescence microscopy. Nuclei were stained blue with DAPI, and HB-EGF-C were stained red with anti-HB-EGF-C antibody. Cells were stimulated with 200 nM TPA for 60 min. **C**) Western blot analysis of EGFR phosphorylation induced by TPA (200 nM) with or without cetuximab (10 μg/ml) in KATO III/wt-HB-EGF and KATO III/HB-EGF-mC.

To examine the role of nuclear HB-EGF-C in gastric cancer cells, we established KATO III stable cell lines that express wt-HB-EGF or HB-EGF-mC. We first evaluated the protein levels of proHB-EGF and HB-EGF-CTF in KATO III/mock, KATO III/wt-HB-EGF cells, and KATO III/HB-EGF-mC cells by Western blotting (Figure 3A). KATO III/wt-HB-EGF cells and KATO III/HB-EGF-mC cells expressed significant levels of proHB-EGF, and proHB-EGF expression decreased and HB-EGF-CTF was generated by shedding of proHB-EGF with 12-*O*-tetradecanoylphorbol-13-acetate (TPA).

We next investigated the intracellular localization of HB-EGF-C in KATO III/wt-HB-EGF cells and KATO III/HB-EGF-mC cells by using an immunofluorescent microscope (Figure 3B). HB-EGF-C translocated from the cytoplasm to the nucleus by adding TPA to activate the processing of proHB-EGF in KATO III/wt-HB-EGF cells, but HB-EGF-C did not accumulate in the nucleus after TPA treatment in KATO III/HB-EGF-mC cells. Meanwhile, TPA induced EGFR phosphorylation in both KATO III/wt-HB-EGF cells and KATO III/HB-EGF-mC cells, and EGFR phosphorylation was inhibited by adding of 10 μg/ml cetuximab (Figure 3C).

We thus confirmed that HB-EGF in KATO III/wt-HB-EGF cells has both functions as an EGFR ligand and HB-EGF-C nuclear translocation, but that of KATO III/HB-EGF-mC has only function as an EGFR ligand without HB-EGF-C nuclear translocation. We also confirmed the same results relating three established forms of MKN45 cells (MKN45/mock, MKN45/wt-HB-EGF, MKN45/HB-EGF-mC) (Data not shown). Using these cell lines, we assessed the behaviors of nuclear HB-EGF-C in gastric cancer cells.

### HB-EGF-C nuclear translocation promotes gastric cancer cell growth

**Figure 4:**
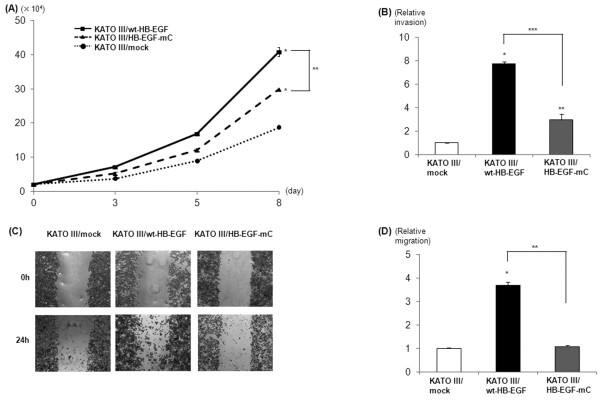
**Cell proliferation and migration in KATO III/mock, KATO III/wt-HB-EGF and KATO III/HB-EGF-mC cells. A**) Cell proliferation assay. Mean of 3 independent clones; bars, SD;* *P* < 0.01, as compared with KATO III/mock; **P < 0.05. **B**) Transwell invasion assay was analyzed in each cell at 48 h after 200 nM TPA stimulation. Value of KATO-III/mock cells was arbitrarily defined as 1. Mean of 3 independent clones; bars, SD;* *P* < 0.01, as compared with KATO III/mock; **P < 0.05, as compared with KATO III/mock; ***P < 0.05. **C**) Wound healing assay. Confluent monolayers of each gastric cancer cells were mechanically wounded with a pipette tip, and photos were obtained at 0 h and 24 h after stimulation of 200 nM TPA (Original magnification: ×40). **D**) Quantification of wound healing assay in 3 independent clones. Migration rate of KATO-III/mock cells at 24 h after TPA stimulation was arbitrarily defined as 1. Mean of 3 independent clones; bars, SD* *P* < 0.01, as compared with KATO III/mock; **P < 0.01.

Cell growth curves in 3 cancer cell lines are shown in Figure 4A. The growth of KATO III/wt-HB-EGF and KATO III/HB-EGF-mC cells was significantly faster than that of KATOIII/mock cells, and the growth of KATO III/HB-EGF-mC cells was significantly decreased compared with KATO III/wt-HB-EGF cells. The same results were observed in three forms of MKN45 cells ( Additional file 1: Figure S1A). These results demonstrate that both conventional signals of HB-EGF as an EGFR ligand and a novel signal for HB-EGF-C nuclear translocation are critical for gastric cancer cell proliferation.

### HB-EGF-C nuclear translocation promotes cell invasion and wound healing

To verify the present results by using clinical samples, we next investigated whether HB-EGF-C nuclear translocation causes an increase in the migration of gastric cancer cells. Three gastric cancer cell lines were used in a transwell invasion assay. As shown in Figure 4B, KATO III/wt-HB-EGF showed a obvious increase in invasion compared with KATO III/mock cells, whereas KATO III/HB-EGF-mC cells did a slight increase. Moreover, KATO III/HB-EGF-mC cells showed a significant decrease in invasion compared with KATO III/wt-HB-EGF cells. Although the differences were smaller in comparison with KATO III cells, the same results were observed in three forms of MKN45 cells ( Additional file 1: Figure S1B).

Wound healing assays have been employed to study cell polarization or tissue matrix remodeling or to estimate cell proliferation and migration rates. HB-EGF is well known to be upregulated in the wound healing process of certain cell types, including keratinocytes [11,12]. We studied the effect of HB-EGF on wound healing by using 3 gastric cancer cell lines (Figure 4C, D). The effectiveness of wound healing was observed in KATO III/wt-HB-EGF, but no significant effect was observed in KATO III/HB-EGF-mC. Although a little wound healing effect was observed in MKN45/HB-EGF-mC, the same tendency as KATO III cells was observed in MKN45 cells ( Additional file 1: Figure S1C, D). These results suggest that HB-EGF-C nuclear translocation rather than HB-EGF as an EGFR ligand is critical for gastric cancer cell migration.

## Discussion

EGFR and EGFR ligands have been extensively studied because the inactivation of EGFR represents a promising strategy for the treatment of several cancers [13]. However, there have been no studies regarding the actions of proHB-EGF and HB-EGF-CTF induced by the cleavage of EGFR ligands. We previously reported that nuclear translocation of HB-EGF-CTF results in a unique signal transduction pathway for cell growth [6,7,10,14]. In the present study, we demonstrated the role of HB-EGF-C in human gastric cancer.

Of the EGFR ligands, HB-EGF is considered the most important growth factor because HB-EGF-knockout mice die shortly after birth, in contrast to the effects of other EGFR ligands [15]. HB-EGF, which is an inducer of tumor growth and angiogenesis, induces resistance to chemotherapy [16,17]. The expression of HB-EGF has been demonstrated in many human cancers, such as hepatocellular carcinoma [18], pancreatic cancer [19], gastric cancer [20,21], colorectal cancer [22], and ovarian cancer [23]. In human gastric cancer, a previous study reported that HB-EGF immunoreactivity was detected in 61 to 72 % of cancer cells and was generally stronger in deeply invasive cancer cells than in cancer cells of more superficial layers [20,21]. Moreover, a recent report suggested that HB-EGF promoted peritoneal carcinomatosis from gastric cancer [24]. HB-EGF is thus considered a potential growth factor in gastric cancer.

In the present study, proHB-EGF expression was observed in 45.8 % of human gastric cancer cases, particularly in deeply invasive gastric cancer. This frequency was slightly lower than the previous reports [20,21], but it is considered that positive for both anti-HB-EGF and anti-HB-EGF-C antibodies was defined as HB-EGF-positive in the present study. Notably, we observed nuclear expression of HB-EGF-C in deep gastric cancers (pT3 and pT4).

In order to confirm the relationship between invasion and HB-EGF-C nuclear localization that had been demonstrated in clinical experiments, we successfully constructed gastric cancer cell lines with and without HB-EGF-C nuclear translocation by overexpressing wt-HB-EGF and HB-EGF-mC. We were thus able to selectively analyze the fundamental effects of HB-EGF-C nuclear translocation *in vitro*.

The present study showed that both the function of HB-EGF as an EGFR ligand and HB-EGF-C nuclear translocation induce gastric cancer growth, whereas HB-EGF-C nuclear translocation independently plays a critical role in gastric cancer invasion. Hence, the present *in vitro* findings support the data of clinical experiments. Recent studies have shown that the inhibition of proHB-EGF shedding promoted cell-cell interactions and decreased cell migration [25,26]. HB-EGF-C signals in the present study may involve these previous results related to cell migration.

Nuclear staining of HB-EGF is reportedly a progressive and poor prognostic factor in bladder cancer [27,28]. Nuclear translocation of proHB-EGF and HB-EGF-CTF might be the factor responsible for this phenomenon because an antibody against HB-EGF-C was used in these studies. Our study showed that nuclear HB-EGF-C is an important progressive factor in gastric cancer as well as in bladder cancer. We cannot precisely elucidate which of proHB-EGF and HB-EGF-CTF has more responsibility for gastric cancer invasion. However, we can speculate that HB-EGF-CTF would be involved more than proHB-EGF, because much HB-EGF-CTF yield after shedding of proHB-EGF that is necessary for HB-EGF-C nuclear translocation. Further investigation is necessary, as the differences between proHB-EGF and HB-EGF-CTF in the nucleus and the detailed mechanisms inducing cell migration are not yet fully understood; clarifying these mechanisms may result in the development of new cancer therapies.

## Conclusions

The present study showed that HB-EGF-C nuclear translocation is involved in gastric cancer development, in addition to the conventional function of HB-EGF as an EGFR ligand. HB-EGF-C nuclear translocation may offer a prognostic marker and a new molecular target for gastric cancer therapy.

## Methods

### Materials

Anti-HB-EGF-C antibody was used to recognize HB-EGF-C, as previously described [7], and anti-HB-EGF antibody (R&D Systems, Minneapolis, MN) was used to recognize the proHB-EGF ectodomain. Cetuximab ((Merk, Darmstadt, Germany)), a monoclonal antibody to EGFR, was used to inhibit EGFR phosphorylation.

### Immunohistochemistry

Immunohistochemical staining was performed using anti-HB-EGF antibody and anti-HB-EGF-C antibody. Consecutive 4-μm-thick sections were deparaffinized and hydrated through a graded series of alcohols. After inhibiting endogenous peroxidase activity by immersion in a 3 % H_2_O_2_/methanol solution, antigen retrieval was achieved by heating the samples in 10 mM citrate buffer (pH 6.0) in a microwave oven for 10 min at 98 °C. Next, sections were incubated overnight with primary antibodies. After thorough washing in PBS (−), the samples were incubated with biotinylated secondary antibodies and then with avidin-biotin-horseradish peroxidase complexes (Vectastain Elite ABC kit; Vector Laboratories, Burlingame, CA). Finally, immune complexes were visualized by incubation in 0.01 % H_2_O_2_ and 0.05 % 3,3′-diaminobenzidine tetrachloride. Nuclear counterstaining was accomplished with Mayer’s hematoxylin.

### Clinical patients and assessment

Using information from a computerized database, we obtained gastric cancer specimens from 96 patients who had undergone surgical operations for gastric adenocarcinoma between January 2002 and December 2006 at the Nagoya City University Hospital. Written general consent that included research uses of clinical data had been obtained from all patients. The study was performed in accordance with the Declaration of Helsinki and Japanese ethical guidelines for epidemiological research. We obtained an institutional review board (IRB) waiver to conduct this study from the chairperson of the IRB. All 96 patients were histologically diagnosed with gastric cancer. Clinical stage was determined according to the Seventh edition of the UICC-TNM classification [29]. All immunostained specimens were assessed by 2 investigators who were blinded to all clinical information. When more than 10 % of the cancer cells in each section were stained for both anti-HB-EGF antibody and anti-HB-EGF-C antibody, immunostaining was defined as positive.

### Cell culture

We used KATO III and MKN45 gastric cancer cell line (Japan Health Science Research Resources Bank, Tokyo, Japan) in our investigation. KATO III and MKN45 cells were maintained in RPMI1640 (Sigma-Aldrich Co., St. Louis, MO) medium that was supplemented with 10 % fetal bovine serum (FBS). Cells were cultured at 37 °C in 5 % CO_2_ humidified air.

### Transfection

cDNAs encoding wild-type HB-EGF (wt-HB-EGF) and the mutant that carry a point mutation at K201A of the C-terminus (HB-EGF-mC) [10] were subcloned into pME18SIII with hygromycin-resistance gene. As previously shown, HB-EGF-mC does not translocate from the plasma membrane to the nucleus after TPA stimulation. All cDNA constructs were verified by DNA sequencing using a CEQ 8000 DNA Analysis System (Beckman Coulter, Brea, CA).

The KATO III human gastric cancer cell line was transfected with wt-HB-EGF (KATO III/wt-HB-EGF) and HB-EGF-mC (KATOIII/HB-EGF-mC) by using Lipofectamine 2000 (Invitrogen, Carlsbad, CA), according to the manufacturer’s instructions, and stably transfected clones were isolated using hygromycin B (Invitrogen). As a control, KATO III was transfected with an empty plasmid (KATO III/mock). As well as KATO III cells, MKN45/wt-HB-EGF, MKN45/HB-EGF-mC and MKN45/mock cells were established. These cells were maintained in RPMI1640 medium that was supplemented with 10 % FBS and 800 μg/mL hygromycin B.

### Immunofluorescence microscopy

Samples were fixed with ethanol and acetone and incubated with primary antibodies against HB-EGF-C. Secondary antibodies was Alexa Fluor® 594 goat anti-rabbit IgG (H + L) (Invitrogen). All sections were counterstained with DAPI (KPL, Inc., Gaithersburg, MD). Images were obtained using an Eclipse 80i fluorescent microscope (Nikon, Tokyo, Japan).

Each gastric cancer cell in a subconfluent state was placed in serum-free medium for 24 h and stimulated with 200 nM TPA (Cell Signaling Technology, Danvers, MA) for 60 min. Cells were stimulated by TPA in order to investigate the localization of HB-EGF-C by shedding proHB-EGF [30], and the intracellular localization of HB-EGF-C was then analyzed by immunofluorescence.

### Western blotting

Cells were washed with PBS and subsequently dissolved in 1× cell lysis buffer (Cell Signaling Technology) containing 20 mM Tris–HCl (pH 7.5), 150 mM NaCl, 1 mM Na_2_EDTA, 1 mM EGTA, 1 % Triton, 2.5 mM sodium pyrophosphate, 1 mM β-glycerophosphate, 1 mM Na_3_VO_4_, and 1 μg/mL leupeptin. After disruption in an ice bath by using a Bio-ruptor sonicator (Cosmo Bio, Tokyo, Japan) for 15 s, lysates were centrifuged at 15,000 rpm for 10 min at 4°C. Each sample was normalized against an equal protein concentration by using a protein assay kit (Bio-Rad Laboratories, Hercules, CA). A equal quantity of 2× sodium dodecyl sulfate-polyacrylamide gel electrophoresis (SDS-PAGE) sample buffer (0.5 mol/L Tris–HCl, pH 7.2, 1 % SDS, 100 mmol/L β-mercaptoethanol, and 0.01 % bromophenol blue) was added to each sample and boiled for 5 min at 100°C. Aliquots of sample were fractionated on 10 or 15 % SDS-PAGE and then electroblotted onto a nitrocellulose membrane. The membrane was blocked with 5 % skimmed milk in PBS for 1 h at room temperature. The membrane was incubated with the primary antibodies for HB-EGF-C, HB-EGF, EGFR (MILLIPORE, Temecula, CA) or phospho-EGFR (MILLIPORE) overnight at 4°C and then washed with 0.05 % Tween 20 in PBS 3 times at 5-min intervals. The membrane was incubated with secondary antibody for 1 h at room temperature, which was followed by 3 washes with 0.05 % Tween 20 in PBS 3 times at 5-min intervals. The membrane was treated with enhanced chemiluminescence detection reagents (ECL; Amersham, Arlington Heights, IL) for 1 min at room temperature and then exposed to scientific imaging films (Eastman Kodak, Rochester, NY). Proteins were visualized as bands on the images. Filters were stripped and reprobed with monoclonal β-actin antibody (Abcam plc, Tokyo, Japan) as an internal control.

### Cell proliferation assay

Proliferation assays were performed as follows. Cells were seeded at 2.0 × 10^4^ cells in the medium with 10 % FBS on 6-cm diameter dishes. Cells were counted on days 3, 5, and 8 by using an Automatic Cell Counter (Millipore, Billerica, MA). Each experiment was conducted independently in cell lines isolated from 3 independent clones.

### Wound healing assay

A wound healing assay was conducted in order to measure cell motility. Cells were grown to confluence in 6-well plates and serum-starved for 24 h, and then a cross-shaped wound was made on the monolayers by using a sterile 200-μL pipette tip. Cells were washed with PBS, placed in the same media with 200nM TPA, and the cross-shaped wound was photographed under microscope at 0 h and 24 h.

### Transwell invasion assay

Cell migration was assessed using the Cell Invasion Assay (CULTREX, Gaithersburg, MD), which consists of a 96-well transwell tissue culture plate with an 8-μm pore size membranes coated with matrigel (top chamber) and a black receiver plate compatible with a 96-well fluorescent plate reader (bottom chamber). After 24 h of serum starvation, cells (5000 cells/well) in serum-free medium with 200nM TPA were placed in the top chamber, and medium containing 10 % FBS was added to the bottom chamber. After 48 h of incubation in CO_2_ at 37°C, the cells that had invaded the bottom chamber were measured according to the manufacturer’s instructions. Each experiment was conducted in cell lines which was isolated from 3 independent clones.

### Statistical analysis

Values are expressed as the mean ± SD. Data were analyzed using χ^2^ test as appropriate. Multiple comparison was done using Games-Howell’s method. *P*-values < 0.05 were considered statistically significant. Data analyses were performed using Dr. SPSS II for Windows release 11.0.1 J software (SPSS Japan, Tokyo, Japan).

## Abbreviations

EGFR: Epidermal growth factor receptor; proHB-EGF: Membrane-anchored heparin-binding epidermal growth factor-like growth factor; HB-EGF-CTF: A carboxy-terminal fragment of heparin-binding epidermal growth factor-like growth factor; HB-EGF-C: The cytoplasmic domain of heparin-binding epidermal growth factor-like growth factor; s-HB-EGF: Soluble heparin-binding epidermal growth factor-like growth factor; PLZF: Promyelocytic leukemia zinc finger; Bcl6: B-cell lymphoma 6; wt-HB-EGF: Wild-type HB-EGF; HB-EGF-mC: HB-EGF at the C-terminus; PLZF: Promyelocytic leukemia zinc finger; KATO III/wt-HB-EGF: KATO III human gastric cancer cell line transfected with wt-HB-EGF; KATOIII/HB-EGF-mC: KATO III human gastric cancer cell line transfected with HB-EGF-mC; KATO III/mock: KATO III human gastric cancer cell line transfected with an empty plasmid; MKN45/wt-HB-EGF: MKN45 human gastric cancer cell line transfected with wt-HB-EGF; MKN45/HB-EGF-mC: MKN45 human gastric cancer cell line transfected with HB-EGF-mC; MKN45/mock: MKN45 human gastric cancer cell line transfected with an empty plasmid; FBS: Fetal bovine serum; TPA: 12-O-tetradecanoylphorbol-13-acetate.

## Competing interests

All the authors declare that we have no competing interests.

## Author’s contributions

Conception and design, TS; Acquisition of data, TS, MY, TM, and TK; Analysis and interpretation of data, TS; Drafting of the manuscript, TS; Revising it critically for important intellectual content, SF and SH; Final approval of the version to be published, TJ; Acquisition of funding, TS; General supervision of research group, SH and TJ. All authors read and approved the final manuscript.

## Pre-publication history

The pre-publication history for this paper can be accessed here:

http://www.biomedcentral.com/1471-2407/12/205/prepub

## Supplementary Material

Additional file 1: Figure S1 Cell proliferation and migration in MKN45/mock, MKN45/wt-HB-EGF and MKN45/HB-EGF-mC cells. A) Cell proliferation assay. Mean of 3 independent clones; bars, SD;* *P* < 0.01, as compared with MKN45/mock; **P < 0.01. B) Transwell invasion assay was analyzed in each cell at 48 h after 200 nM TPA stimulation. Value of MKN45/mock cells was arbitrarily defined as 1. Mean of 3 independent clones; bars, SD;* *P* < 0.01, as compared with KATO III/mock; **P < 0.05, as compared with KATO III/mock; ***P < 0.05. C) Wound healing assay. Confluent monolayers of each gastric cancer cells were mechanically wounded with a pipette tip, and photos were obtained at 0 h and 24 h after stimulation of 200 nM TPA (Original magnification: ×40). D) Quantification of wound healing assay in 3 independent clones. Migration rate of MKN45/mock cells at 24 h after TPA stimulation was arbitrarily defined as 1. Mean of 3 independent clones; bars, SD* *P* < 0.01, as compared with MKN45/mock; **P < 0.05, as compared with MKN45/mock; ***P < 0.01. Click here for file
