# Supplementary figures and images for: Nuclear translocation of the cytoplasmic domain of HB-EGF induces gastric cancer invasion
Source: BMC Cancer. 2012 May 30;12:205. doi: 10.1186/1471-2407-12-205 (PMC3414754; doi:10.1186/1471-2407-12-205)

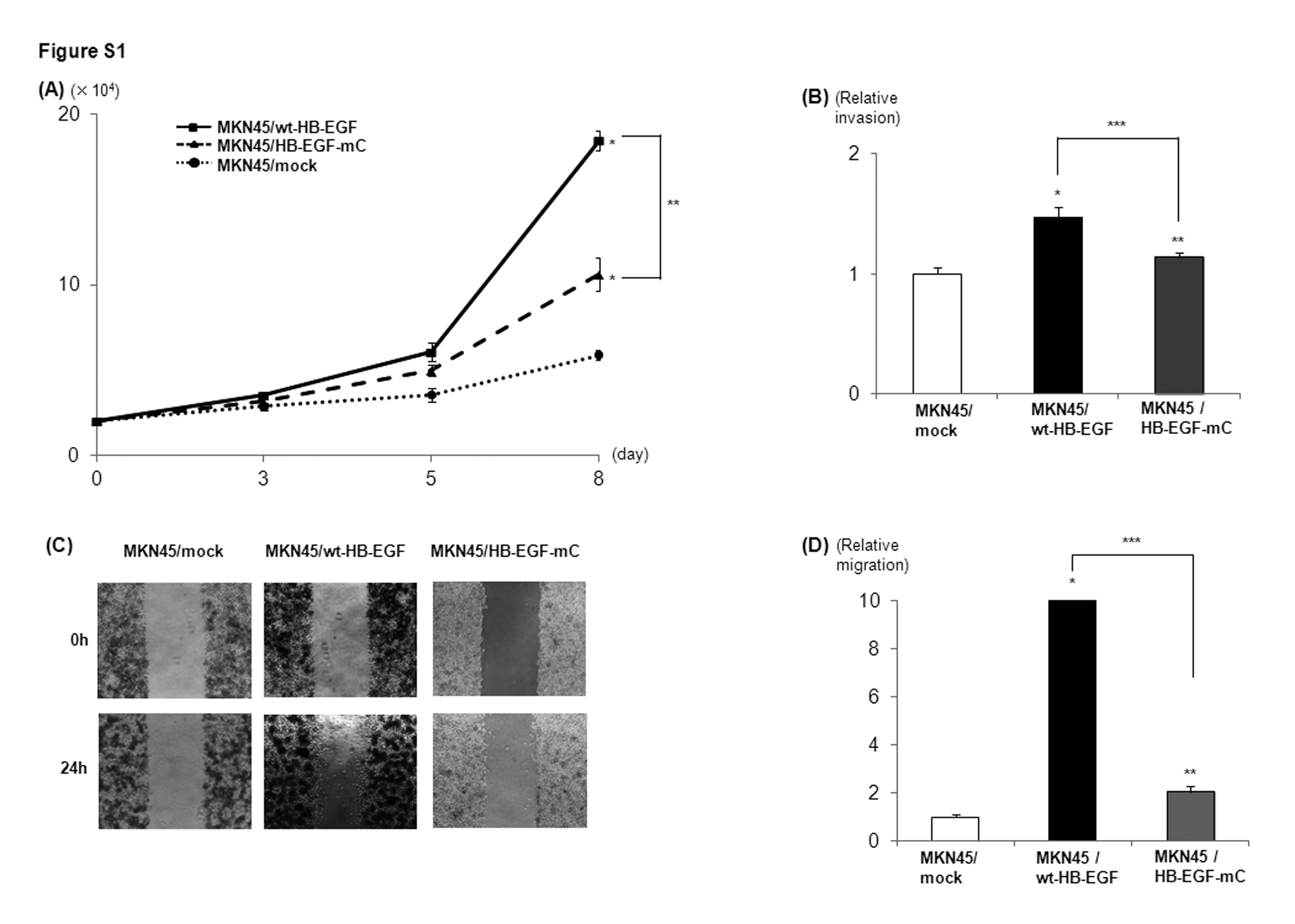

Supplement: Additional file 1: Figure S1 — Cell proliferation and migration in MKN45/mock, MKN45/wt-HB-EGF and MKN45/HB-EGF-mC cells. A) Cell proliferation assay. Mean of 3 independent clones; bars, SD;* P < 0.01, as compared with MKN45/mock; **P < 0.01. B) Transwell invasion assay was analyzed in each cell at 48 h after 200 nM TPA stimulation. Value of MKN45/mock cells was arbitrarily defined as 1. Mean of 3 independent clones; bars, SD;* P < 0.01, as compared with KATO III/mock; **P < 0.05, as compared with KATO III/mock; ***P < 0.05. C) Wound healing assay. Confluent monolayers of each gastric cancer cells were mechanically wounded with a pipette tip, and photos were obtained at 0 h and 24 h after stimulation of 200 nM TPA (Original magnification: ×40). D) Quantification of wound healing assay in 3 independent clones. Migration rate of MKN45/mock cells at 24 h after TPA stimulation was arbitrarily defined as 1. Mean of 3 independent clones; bars, SD* P < 0.01, as compared with MKN45/mock; **P < 0.05, as compared with MKN45/mock; ***P < 0.01. [file 1471-2407-12-205-S1.tiff]
